# 

**Published:** 1875-02

**Authors:** 


CONTENTS:

| | PAGE |
|---|---|
| Diptheria | 37 |
| Inhaling Bad Air | 39 |
| Keep Your Feet Warm | 40 |
| Our Summer at Maplewood | 41 |
| How it all Happened | 42 |
| Sleeplessness | 44 |
| Condemned Food | 45 |
| Feeble Females | 46 |
| More Light Sought | 47 |
| Indigestible Substances | 48 |
| The Fool-Killer | 49 |
| Strange, if True | 50 |
| Soft Food | 51 |
| Convalescence | 52 |
| Scientific Investigation | 53 |
| Cosmetics | 54 |
| Female Education | 55 |
| A Trying Time | 56 |
| Disposing of the Dead | 58 |
| Purifying the Skin | 60 |
| Huddling Human Beings | 61 |
| The Vice of Parsimony | 61 |
| Encourage the Boys | 62 |
| Furnace Heat | 63 |
| The House of Steinway | 64 |
| The Best Dentifrice | 65 |
| Ornament and Use | 65 |
| The Economic | 66 |
| Malignant Throat Diseases | 67 |
| The Air we Breathe | 68 |

